# One‐year follow‐up of survival and health‐related quality of life in patients with medical conditions admitted acutely to hospital in Malawi and Tanzania

**DOI:** 10.1111/anae.70083

**Published:** 2025-11-14

**Authors:** Nateiya M. Yongolo, Ibrahim G. Simiyu, Stephen A. Spencer, Eve Worrall, Ben Morton, the Multilink Consortium

**Affiliations:** ^1^ Liverpool School of Tropical Medicine Liverpool UK

Multimorbidity, defined as the coexistence of two or more long‐term conditions, is a major public health issue. In southern and eastern Africa, limited access to primary care frequently results in delayed diagnosis and chronic disease decompensation with the need for acute hospital admission [1]. This report details 1‐year survival and health‐related quality of life (HRQoL) data in patients admitted acutely to hospitals in Malawi and Tanzania and follows on from our study that detailed the high prevalence of hypertension (44%); HIV (31%); and diabetes mellitus (27%) in patients admitted to hospital [2].

We conducted this study in line with our published protocol [3] and with statistical methodology replicated from our multicentre cohort study [2]. Patients were categorised based on the number (none, one and two or more) of long‐term conditions. The 1‐year follow‐up for all patients was completed in December 2024. We evaluated 1‐year survival and HRQoL (where a score of 1 represents perfect health and a score of 0 represents death) in survivors using EQ5D‐5l. We have provided an open access link to our database hosted at: https://publications.mlw.mw. The 1‐year survival analysis for this study was performed in R (4.4.1; R Studio for Statistical Computing, Vienna, Austria) using the survival, survminer and ggplot2 packages and HRQoL analysis was conducted in Stata MP 18.0 (StataCorp, College Station, TX, USA).

A total of 1407 patients, 657 (47%) female, were recruited to the cohort study between September 2022 and July 2023. One‐year outcomes were available for 1244 (88%) patients, with 551 (44%) deaths recorded. There were 161 (11%) patients lost to follow‐up at 1 year, and two patients withdrew from the study. There were 416 (54%) deaths at 1 year among those with two or more long‐term conditions (Table 1 and online Supporting Information Figure S1). After adjusting for age, sex, universal vital assessment (a context‐sensitive early warning score [4]) and site, patients with two or more long‐term conditions had a higher risk of mortality compared with those with one long‐term condition (hazard ratio 1.89, 95%CI 1.32–2.70) (Fig. 1, online Supporting Information Tables S1 and S2).

**Table 1:** Crude patient mortality across the follow‐up period by the number of long‐term conditions.

Long‐term conditions	Inpatient mortality	30‐day mortality	90‐day mortality	1‐year mortality
0	14 (6%)	21 (9%)	31 (14%)	39 (18%)
1	31 (11%)	52 (18%)	80 (28%)	99 (38%)
≥ 2	142 (18%)	249 (30%)	335 (42%)	413 (54%)
Total	187 (14%)*	322 (24%)*	446 (34%)*	551 (44%)

* Denominators differ due to non‐attendance at follow‐up appointments or loss to follow‐up. Outcome status was not captured for: 95 (7%) patients at hospital discharge; 41 (3%) at day 30; 90 (6%) at day 90; and 163 (12%) at 1‐year.

**Figure 1:**
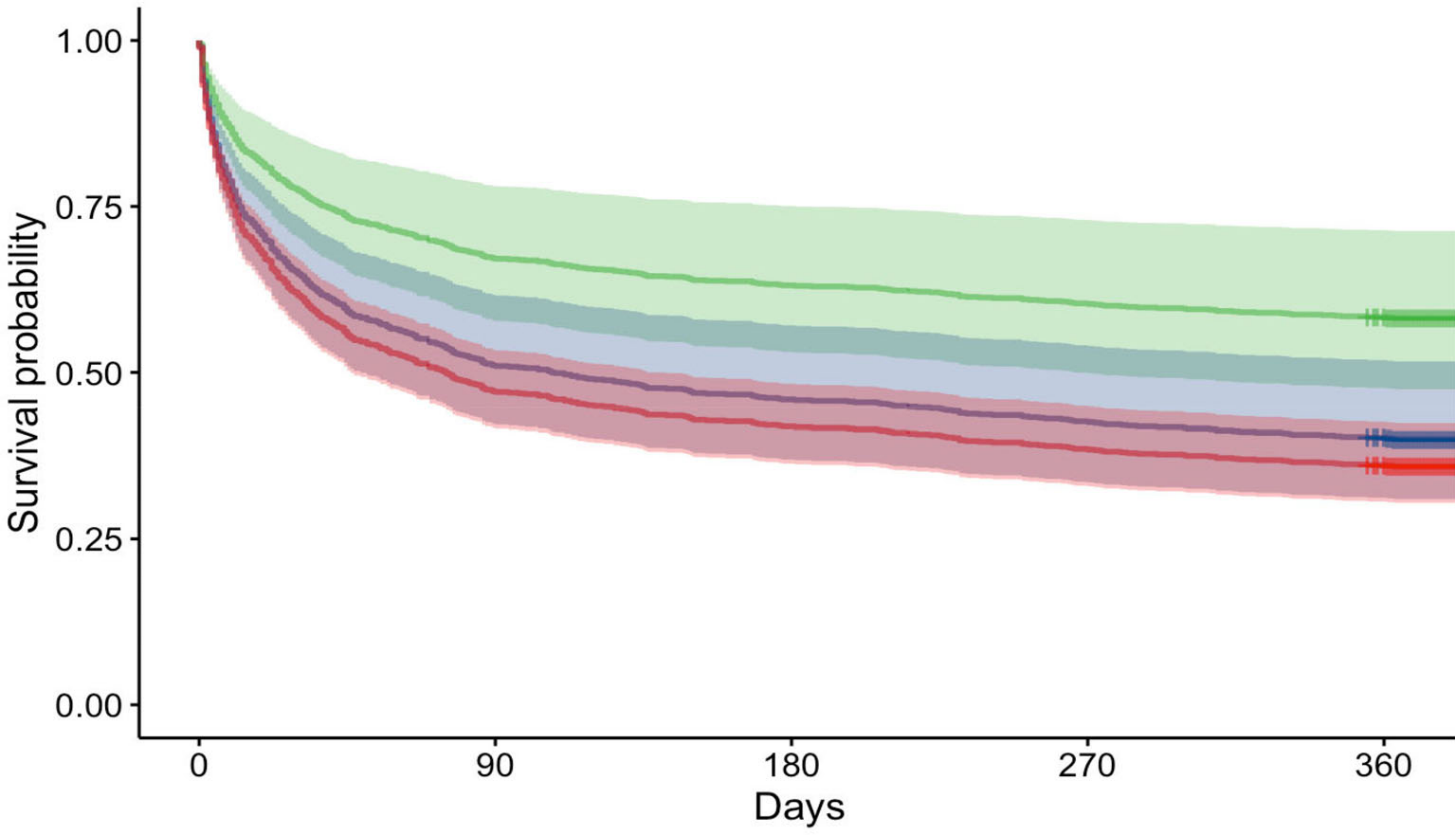
Kaplan–Meier survival 1 year after hospital admission by number of long‐term conditions. Adjusted survival‐curve, based on Cox regression model adjusted for age, sex, universal vital assessment and site. Vertical drops represent censored patients (alive or loss to follow‐up at their last contact). The number at risk, events and survival probability are shown in online Supporting Table S3. No long‐term conditions, n = 242; single long‐term condition, n = 300; and ≥ 2 long‐term conditions, n = 865.

In the unadjusted analysis, the adjusted median (IQR [range]) 1‐year HRQoL was lower in patients with two or more long‐term conditions compared with those with one long‐term condition (0.783 (0.625–1.000 [‐0.743–1.000]) vs. 1.000 (0.799–1.000 [‐1.069–1.000]), p < 0.001). After adjustment, however, this difference was not statistically significant: coefficient ‐0.02, 95%CI ‐0.04 to ‐0.003, p < 0.095 (online Supporting Information Figure S2 and Table S3).

We found lower survival for patients with long‐term conditions, most commonly HIV, hypertension and diabetes mellitus. This is important because complications from these primary chronic communicable and non‐communicable diseases could potentially be mitigated with health systems strengthening before secondary complications occur. For example, we found a high prevalence of heart failure (11%); cerebral vascular accident (9%); and chronic kidney disease (6%) in our cohort study [2]. The question of how best to support health systems and healthcare workers to deliver these interventions (e.g. improved diagnostics, drugs and patient‐centred care) is a key question that needs to be addressed. We recommend that integrated care programmes be developed for hospitalised patients in these settings, building on the established vertical health systems for patients living with HIV.

To our knowledge, this is the first examination of long‐term outcomes for patients admitted to hospital with multimorbidity in southern and eastern Africa. Our findings contrast with data from high‐income countries, which report higher risk mortality (6% vs. 3% in‐hospital mortality) and readmissions (39% vs. 25% at 1 year) among patients with multimorbidity [5]. Our HRQoL data also align with previous studies that report dose‐dependent reductions in HRQoL as the number of conditions increases [6, 7] 90 days after admission but this effect was not observed in survivors at 1 year. In resource‐constrained health systems like those in Tanzania and Malawi, responding to this challenge requires a dual focus to provide appropriate care for those already affected, whilst prioritising primary preventative strategies in primary healthcare settings to promote population health [8].

Multimorbidity is associated with poor outcomes for hospitalised patients in southern and eastern Africa, with persistent increased mortality 1 year after admission. Future research should identify locally relevant risk profiles and test health system‐level interventions to better control chronic disease, prevent acute decompensation and mitigate long‐term health consequences of multimorbidity.

## Supporting information


**Figure S1.** One‐year survival after hospital admission crude Kaplan–Meier plot by number of long‐term conditions.
**Figure S2.** Health‐related quality of life utility scores at baseline and among survivors at the day 90‐ and 1‐year observation.

**Table S1.** One‐year survival after hospital admission: crude and adjusted variable analysis.
**Table S2.** Mortality risk by number of long‐term conditions at 90 and 365 days after hospital admission.
**Table S3.** Association between the number of long‐term conditions and health‐related quality of life utility scores 1‐year after hospital admission.
