# Supplementary material for: One‐year follow‐up of survival and health‐related quality of life in patients with medical conditions admitted acutely to hospital in Malawi and Tanzania
Source: Anaesthesia. 2025 Nov 14;81(2):297–9. doi: 10.1111/anae.70083 (PMC12803564; doi:10.1111/anae.70083)

Figure S1: One-year survival after hospital admission crude Kaplan-Meier plot by number of long-term conditions

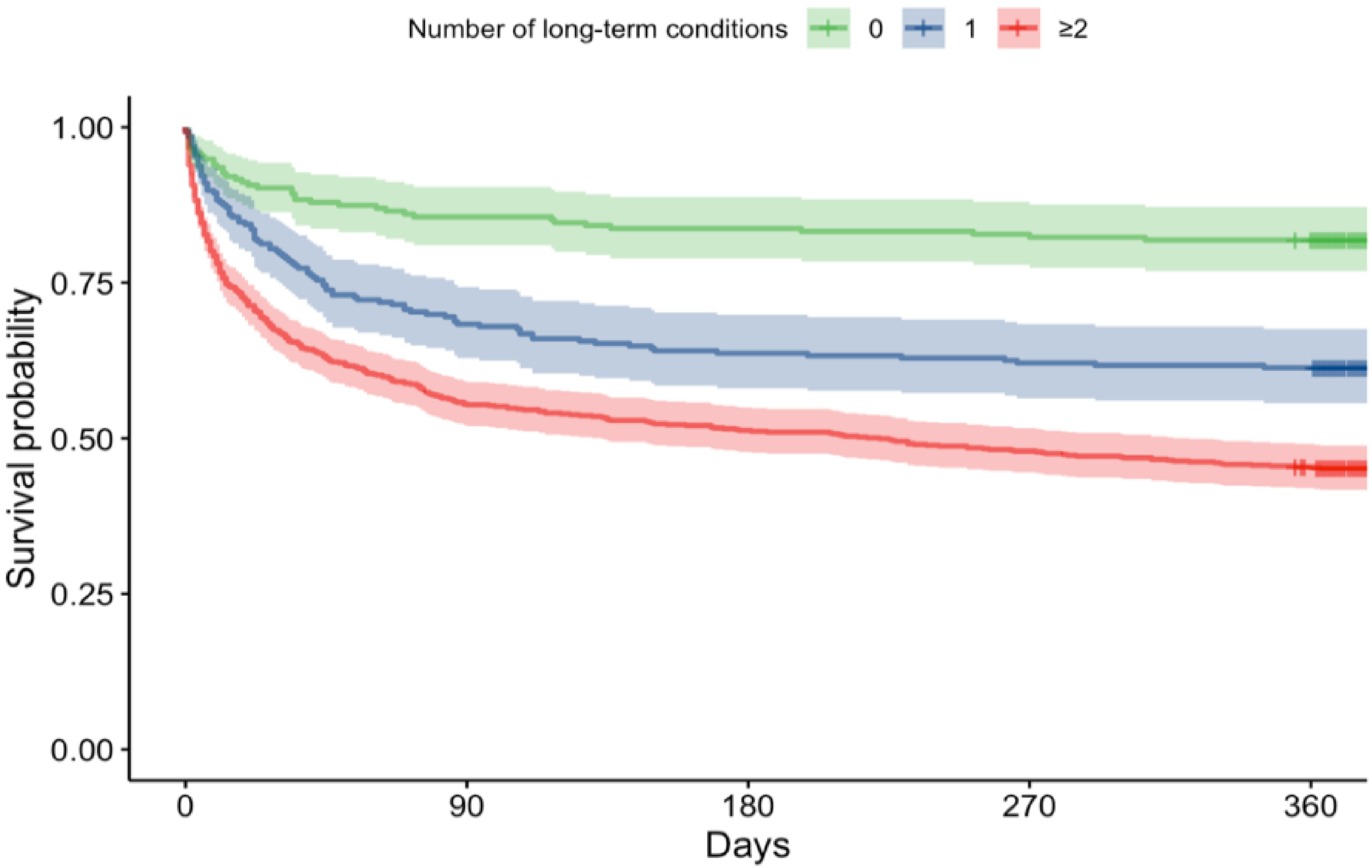

Figure S2: Box and whisker plot of health-related quality of life (HRQoL) utility scores at baseline, and among survivors at the day 90- and one-year observation; p-values reflect multivariable GLM analyses (gamma distribution), adjusted by age, sex, site, universal vital assessment (baseline); and age, sex, site, number of days in study (day 90 and 1 year).

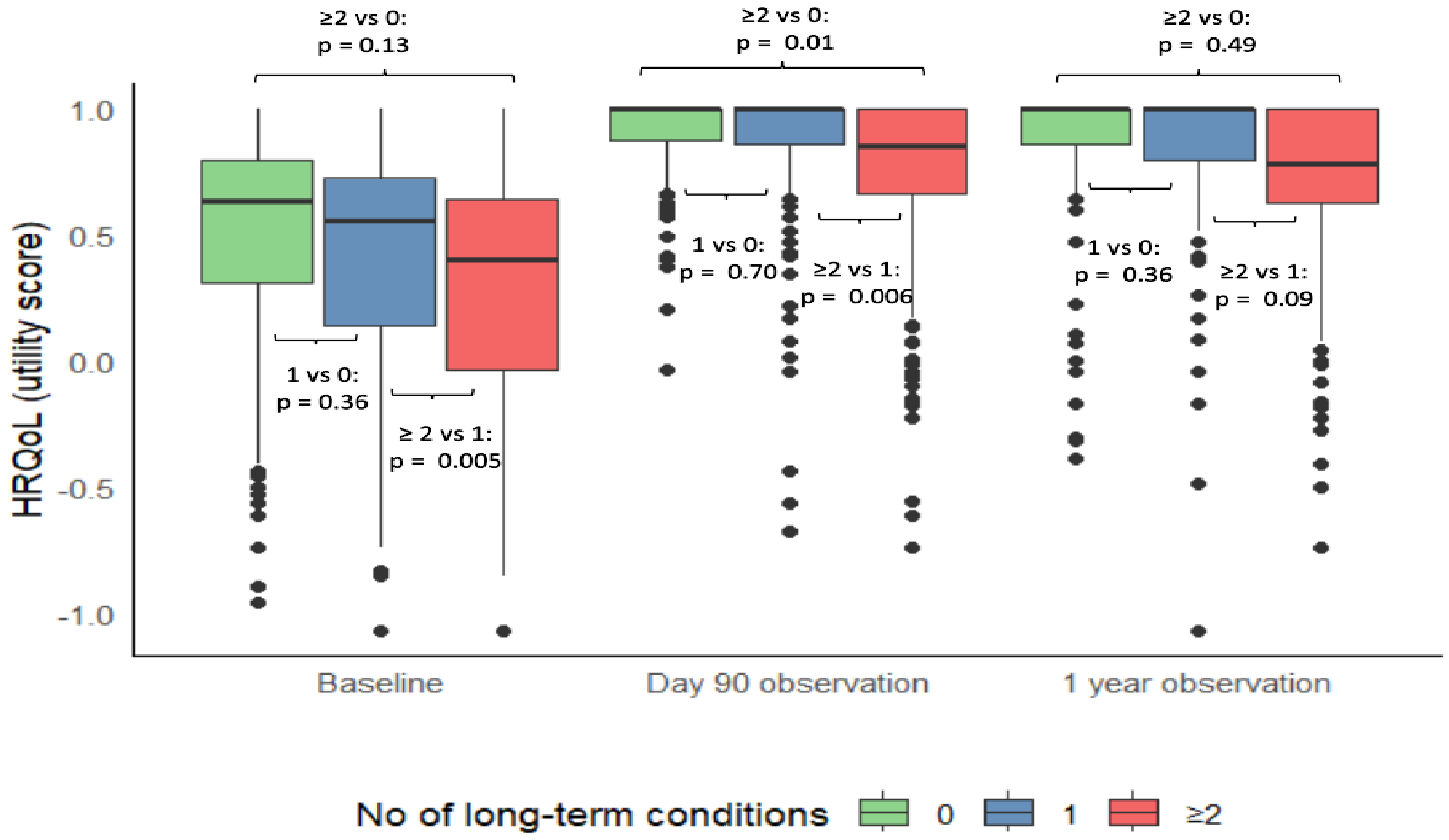

Supplement: Supplementary file 1 — Figure S1. One‐year survival after hospital admission crude Kaplan–Meier plot by number of long‐term conditions. Figure S2. Health‐related quality of life utility scores at baseline and among survivors at the day 90‐ and 1‐year observation. [file ANAE-81-297-s002.pdf]
