# Supplementary material for: One‐year follow‐up of survival and health‐related quality of life in patients with medical conditions admitted acutely to hospital in Malawi and Tanzania
Source: Anaesthesia. 2025 Nov 14;81(2):297–9. doi: 10.1111/anae.70083 (PMC12803564; doi:10.1111/anae.70083)
Supplement: Supplementary file 2 — Table S1. One‐year survival after hospital admission: crude and adjusted variable analysis. Table S2. Mortality risk by number of long‐term conditions at 90 and 365 days after hospital admission. Table S3. Association between the number of long‐term conditions and health‐related quality of life utility scores 1‐year after hospital admission. [file ANAE-81-297-s001.docx]

|  | **Unadjusted** | | | | **Adjusted** | | |
| --- | --- | --- | --- | --- | --- | --- | --- |
| **Characteristic** | **N** | **HR** | **95% CI** | **p-value** | **HR** | **95% CI** | **p-value** |
| Number of long-term conditions | 1224 |  |  |  |  |  |  |
| 0 |  | — | — |  | — | — |  |
| 1 |  | 2.41 | 1.66, 3.48 | <0.001 | 1.69 | 1.16, 2.47 | 0.006 |
| ≥2 |  | 3.93 | 2.83, 5.46 | <0.001 | 1.89 | 1.32, 2.70 | <0.001 |
| Age | 1224 | 1.02 | 1.02, 1.03 | <0.001 | 1.02 | 1.01, 1.02 | <0.001 |
| Sex | 1224 |  |  |  |  |  |  |
| Female |  | — | — |  | — | — |  |
| Male |  | 1.12 | 0.94, 1.32 | 0.2 | 1.24 | 1.04, 1.48 | 0.015 |
| Universal vital assessment | 1224 | 1.23 | 1.18,1.27 | <0.001 | 1.20 | 1.15,1.24 | <0.001 |
| Hospital | 1224 |  |  |  |  |  |  |
| Chiradzulu District Hospital |  | — | — |  | — | — |  |
| Hai District Hospital |  | 1.13 | 0.86, 1.48 | 0.4 | 1.01 | 0.76, 1.34 | >0.9 |
| Muhimbili national Hospital |  | 2.21 | 1.75, 2.79 | <0.001 | 1.70 | 1.32, 2.19 | <0.001 |
| Queen Elizabeth Hospital |  | 0.90 | 0.70, 1.16 | 0.4 | 0.95 | 0.74, 1.23 | 0.7 |
| Abbreviations: CI = Confidence Interval, HR = Hazard Ratio  183 observations deleted due to missingness | | | | | | | |

**Table S1**: One year survival after hospital admission: crude and adjusted variable analysis.

**Table S2:** Mortality risk by number of long-term conditions at 90 and 365 days after hospital admission.

| **0 long-term condition** | | | | | |
| --- | --- | --- | --- | --- | --- |
| Time  (days) | At Risk | Events | Survival probability % | Standard error | 95% CI |
| 90 | 184 | 31 | 85.6 | 0.02 | 81.0-90.4 |
| 365 | 165 | 8 | 81.9 | 0.03 | 76.9-87.2 |
| **1 long-term condition** | | | | | |
| 90 | 175 | 81 | 68.4 | 0.02 | 62.9-74.3 |
| 365 | 148 | 18 | 61.3 | 0.03 | 55.6-76.6 |
| **≥ 2 long-term conditions** | | | | | |
| 90 | 418 | 336 | 55.4 | 0.02 | 51.9-59.0 |
| 365 | 328 | 77 | 45.1 | 0.02 | 41.7-48.8 |

**Table S3**: Association between the number of long-term conditions and health related quality of life (HRQoL) utility scores one year after hospital admission

|  | Total, N=661 | No long-term conditions,  N = 174 | 1 long-term condition,  N = 153 | ≥2 long-term conditions,  N = 334 | | 1 vs no long-term conditions:  p-value | | | | ≥2 vs no long-term conditions:  p-value | | | | ≥2 vs 1 long-term conditions:  p-value | | | |  |
| --- | --- | --- | --- | --- | --- | --- | --- | --- | --- | --- | --- | --- | --- | --- | --- | --- | --- | --- |
|  |  |  |  |  | | Coefficient (95% CI) | | p-value | | Coefficient (95% CI) | | p-value | | Coefficient (95% CI) | | p-value | |  |
| Year1 observation median HRQoL health utility (IQR) | 0.927 (0.716, 1.00) | 1.000 (0.862, 1.000) | 1.000 (0.799, 1.000) | 0.783 (0.625, 1.000) | | NA | | 0.462^§^ | | NA | | **<0.0001^§^** | | NA | | **<0.0001^§^** | |  |
|  |  |  |  |  | | 0.01 (-0.01, 0.03) | | 0.3594* | | -0.01 (-0.03, -0.01) | | 0.494* | | -0.02 (-0.04, -0.003) | | 0.095* | |  |
|  |  |  |  |  | |  | |  | |  | |  | |  | |  | |  |
|  |  |  |  |  | | *0.01 (-0.01, 0.03)* | | *0.554^‡^* | | *-0.01 (-0.03, -0.005)* | | *0.140^‡^* | | *-0.02 (-0.04, -0.001)* | | *0.039^‡^* | |  |
| *Sensitivity analysis: depression excluded from multimorbidity disease count* | | | | |  | |  | |  | |  | |  | |  | |  | |
|  | *N = 661* | *N = 179* | *N = 164* | *N=318* | |  | |  | |  | |  | |  | |  | |  |
| *Year1 observation median HRQoL health utility (IQR)* | *0.927 (0.716, 1.000)* | *1.000 (0.859, 1.000)* | *1.000 (0.792, 1.000)* | *0.781 (0.625, 1.000)* | | *NA* | | *0.362^§^* | | *NA* | | ***<0.0001^§^*** | | *NA* | | ***<0.0001^§^*** | |  |
|  |  |  |  |  | | *0.02 (-0.005, 0.04)* | | *0.136** | | *0.002 (-0.02, -0.02)* | | *0.875** | | *-0.01 (-0.03, -0.005)* | | *0.162** | |  |
|  |  |  |  |  |  |  |  |  |  |  |  |  |  |  |  |  |  |  |

^§^Mann-Whitney U test

*GLM (Gamma distribution) multivariable model with adjustments for age, sex, number of days between admission, and site

*Italic fonts reflect sensitivity analyses.*

^‡^ Multiple imputation estimates based on GLM (Gamma distribution) multivariable model with adjustments for age, sex, site, and number of days between admission and 1 year observation. Multiple imputation with chained equations (MICE; *m* = 10 imputed datasets). The MI analyses were conducted using all the variables in the analysis model (HRQoL utility at 1 year, number of long-term conditions, age, sex, site, number of days between admission and year 1 observation), as well as the auxiliary variables (HRQoL utility at baseline, disability at baseline, clinical frailty, mid-upper arm circumference, and individual diseases: hypertension, diabetes, CKD, depression, HIV, heart failure, stroke, chronic liver disease, ischaemic heart disease, COPD). The variables with imputed data included: HRQoL utility at the year 1 observation. The imputation was conducted using STATA 18 (*mi impute chained* command); 10 datasets were imputed and with a burn-in of 10 iterations.

NA: not applicable
